# Using hand grip strength to detect slow walking speed in older adults: the Yilan study

**DOI:** 10.1186/s12877-021-02361-0

**Published:** 2021-07-16

**Authors:** Yen-Huai Lin, Hsi-Chung Chen, Nai-Wei Hsu, Pesus Chou

**Affiliations:** 1 Department of Medical Imaging, Cheng Hsin General Hospital, Taipei, Taiwan; 2 Department of Medicine, School of Medicine, National Yang Ming Chiao Tung University, Taipei, Taiwan; 3 Department of Psychiatry and Center of Sleep Disorders, National Taiwan University Hospital, Taipei, Taiwan; 4 Community Medicine Research Center, National Yang Ming Chiao Tung University, Taipei, Taiwan; 5 Division of Cardiology, Department of Internal Medicine, National Yang Ming Chiao Tung University Hospital, 152 Hsing-Ming Road, 26042 Yilan, Taiwan; 6 Public Health Bureau, Yilan County, Taiwan

**Keywords:** Hand grip strength, Walking speed, Yilan study

## Abstract

**Background:**

Walking speed is an important health indicator in older adults, although its measurement can be challenging because of the functional decline due to aging and limited environment. The aim of this study was to examine whether hand grip strength can be a useful proxy for detecting slow walking speed in this population.

**Methods:**

A cross-sectional study was conducted using the cohort from the Yilan Study in Taiwan. Community-dwelling older adults aged 65 years and older were included. Slow walking speed was defined as a 6-meter walking speed < 1.0 m/s, according to the 2019 Asian Working Group for Sarcopenia diagnostic criteria. Stepwise multiple linear regression was used to determine the most significant variables associated with walking speed. Receiver operating characteristic analysis was used to determine the optimal cutoff values for hand grip strength in detecting slow walking speed.

**Results:**

A total of 301 participants with an average age of 73.9 ± 6.8 years were included; 55.1 % participants were women. In stepwise multiple linear regression analysis that included various variables, hand grip strength was found to be the most explainable factor associated with walking speed among all participants and among participants of each sex. The optimal cutoff values for hand grip strength in the detection of slow walking speed were 19.73 kg for all participants (sensitivity: 55 %, specificity: 83 %, area under the curve: 0.74, accuracy: 66.9 %), 35.10 kg for men (sensitivity: 92 %, specificity: 42 %, area under the curve: 0.70, accuracy: 66.4 %), and 17.93 kg for women (sensitivity: 62 %, specificity: 80 %, area under the curve: 0.76, accuracy: 67.9 %).

**Conclusions:**

Hand grip strength was found to be a useful proxy for the identification of slow walking speed in older adults.

## Introduction

The global trend of population aging is increasing dramatically, and frailty has become a vital issue owing to its increasing prevalence and adverse impact on older adults. Frailty is not only associated with hospitalization and institutionalization [1, 2] but it is also a predictor of mortality [1, 3]. Thus, the evaluation of frailty among community-dwelling older adults is essential for public health [4].

In the definition of frailty, hand grip strength and walking speed are the core criteria [5]. Both hand grip strength and gait are altered with aging and reflect the severity of sarcopenia associated with frailty. Compared with hand grip strength, walking speed is specifically used to investigate the decline in mobility [6]. Converging evidence supports the use of walking speed as a component of frailty assessment [7–9]. Therefore, a walking speed of 0.8 m/s is chosen as the cutoff in the European consensus definition of sarcopenia, which is also considered a slow walking speed [10]. There are multiple walking speed tests comprising various distances, including 8, 10, and 15 feet and 3, 4, 5, 6, 11, and 30 m [11, 12]. Among these, the 4- and 6-meter walking speed tests are the most commonly recommended [10, 13]. However, the living environment and decreased cognitive and physical abilities of older adults tend to compromise the feasibility of measuring walking speed, particularly in frail individuals. Therefore, it is important to develop a proxy for the identification of slow walking speed for assessing frailty.

Hand grip strength is another major component in frailty assessment; it is a useful indicator of the current health status. For example, hand grip strength is related to lower bone mineral density or osteoporosis [14, 15], malnutrition [16], cognitive impairment [17], multimorbidity [18], depression [19], frailty [4], and long sleep duration [20]. Thus, owing to the high clinical relevance, availability, and accessibility of measuring hand grip strength, it is intuitive to consider the use of hand grip strength as a proxy for the detection of slow walking speed [21–25].

In the literature, hand grip strength has been proposed as a method to identify slow walking speed (< 0.8 m/s), although the cutoff values varied according to sex and race [21–25]. In a large, diverse sample of older adults, a grip strength of less than 16 kg in women and 26 kg in men was associated with slow walking speed [21]. The Health and Retirement Study found the hand grip strength cutoff values for the detection of slow walking speed to be similar for black and white men (< 35 kg), but higher for black women than for white women (< 31 kg vs. < 22 kg) [23]. In a Turkish study that included 406 older adults, a hand grip strength cutoff value of 22 kg for women had a sensitivity of 76.9 % and specificity of 62.5 %, while in men, a cutoff value of 32 kg had a sensitivity of 80.5 % and specificity of 76.2 % [22]. However, participants in this study were recruited among patients visiting an outpatient clinic and were not community-dwelling older adults, which limits its generalizability. In the InCHIANTI study, a hand grip strength cutoff value of 19.3 kg had an area under the curve (AUC) of 0.90 in women, while a cutoff value of 30.3 kg had an AUC of 0.90 in men [24]. However, this study included participants from the general population, with ages ranging from 20 to 102 years, and not from the older adult population. In a cross-sectional study that included 5783 older adults, a hand grip strength cutoff value of 21 kg for women had a sensitivity of 58.6 %, a specificity of 72.9 % and an AUC of 0.83, whereas a cutoff value of 32 kg for men had a sensitivity of 49.1 %, a specificity of 79.8 %, and an AUC of 0.82 [25]. However, this study included a non-Asian population. To the best of our knowledge, only a few studies have reported cutoff values for hand grip strength to detect slow walking speed among community-dwelling or older Asian adults [22].

Furthermore, in 2019, the Asian Working Group for Sarcopenia (AWGS) updated the diagnostic cutoff value of slow walking speed from ≤ 0.8 to < 1.0 m/s [13]. It is unknown whether hand grip strength is the best proxy for the detection of slow walking speed. Hence, there is a need to examine the magnitude of association between hand grip strength and various potential candidate variables with walking speed. In addition, if appropriate, it is also necessary to determine the optimal cutoff value for hand grip strength based on the most recent definition of slow walking speed.

Therefore, the primary aim of this study was to determine whether hand grip strength is the most optimal explainable correlate for walking speed in Asian community-dwelling older adults compared with various candidate variables and the extent to which hand grip strength correlated with walking speed. After confirming this, the secondary aim of this study was to determine the optimal cutoff values for hand grip strength to detect slow walking speed according to the updated criteria of the AWGS in 2019.

## Methods

### Participants

This cross-sectional study was part of the Yilan Study, an integrative community-based health survey conducted from 2012 to 2016 in Taiwan. The study design has been described in detail elsewhere [26]. Briefly, residents of Yilan City aged 65 years and above were randomly recruited for participation. Well-trained project assistants conducted face-to-face interviews with the participants at their homes. To identify the potential variable and compare their association with walking speed in contrast to hand grip strength, this study included not only sociodemographic characteristics but also smoking and drinking status, lifestyle, physical and mental conditions, and anthropometric measurements in the analysis. The inclusion criteria were the ability to complete the interview and perform the hand grip strength test and 6-meter walking test. We excluded participants who could not complete the interview, were unable to cooperate with the anthropometric measurements because of physical and cognitive disability, or could not perform the 6-meter walking test because of the limited living environment at their homes. Finally, a total of 301 participants (mean age: 74.3 years; range: 65–99 years), who provided written informed consent, were included. This study was approved by the institutional review board of NYMUH (IRB No. 2011A016).

### Measurements of hand grip strength and muscle mass

Hand grip strength (kg) was measured using a digital hand dynamometer (EH101; Camry®, Guangdong Province, China). The grip strength of each hand was assessed twice; the maximal value of each hand was averaged as the final estimate of hand grip strength for analysis. The muscle masses of the trunk, upper and lower extremities were measured using bioelectrical impedance analysis (InnerScan V, TANITA® BC601, Japan).

### Slow walking speed

Participants were requested to walk for 6 m at their usual pace, with additional 2-meter sections for acceleration and deceleration to ensure a consistent walking speed over the measured distance. Slow walking speed was defined as a walking speed < 1.0 m/s, as per the 2019 recommendations of the AWGS [13].

### Other covariates

Data on sociodemographic characteristics including age (≥ 75 vs. < 75 years), sex, body mass index, education status (junior and above vs. primary), and living status (living alone vs. living with others) were obtained. In addition, data on smoking history, drinking status, and frequency of exercise (< 3 vs. ≥ 3 times per week) were recorded. With respect to chronic medical morbidities, self-reported data on diabetes mellitus, hypertension, heart disease, hyperlipidemia, stroke, cancer, gout, respiratory diseases, lower limb arthritis, and falls were collected. Medical diseases were recorded only for participants who reported being diagnosed with a disease and treated for it. The Hospital Anxiety and Depression Scale was used to assess symptoms of depression and anxiety in all participants. The optimal cutoff points for anxiety and depression are 3 and 6 on the respective subscales [27].

### Statistical analysis

The independent *t-test* and one-way analysis of variance were used to examine the factors associated with 6-meter walking speed. Pearson’s correlation coefficient was calculated to investigate the relationship between walking speed, anthropometric measurements, and hand grip strength. Significant variables were included in the stepwise multiple linear regression analysis to determine the variables that significantly correlated with walking speed. The Shapiro-Wilk test was used to confirm the normal distribution of unstandardized residuals (*p* > 0.05). Receiver operating characteristic (ROC) curves and the corresponding AUC were used to evaluate how well hand grip strength was associated with slow walking speed. Youden’s index (sensitivity + specificity − 1) from the ROC curve was used to determine cutoff values. We also compared the performance of hand grip strength as a single proxy with the predicted model using stepwise multiple linear regression to detect slow walking speed. Missing values were regarded as missing completely at random; thus, available-case analyses were implemented to handle missing data. All reported *p*-values were two sided, and the statistical significance level was set at *p* < 0.05. Analyses were performed using SPSS for Windows (version 19.0; IBM Corp., Armonk, NY, USA).

## Results

Table 1 presents the socio-demographic characteristics and lifestyle data associated with the 6-meter walking speed. Participants who were older, were women, had a low education level, and did not exercise regularly had a slower walking speed than other participants. Table 2 presents the physical and mental conditions and their associations with 6-meter walking speed. Participants with diabetes, hypertension, heart disease, stroke, lower limb arthritis, and depression had a slower walking speed than other participants.

**Table 1:** Six-meter walking speed by sociodemographic and lifestyle of participants (*n*=301)

	Total	6-Meter Walking Speed (m/sec)	*p-*value for t-test/ ANOVA
	n (%)	Mean (SD)	
Age (years)			
≥ 75	127 (42.2)	0.79 (0.29)	<0.001
< 75	174 (57.8)	1.03 (0.31)	
Sex			
Women	166 (55.1)	0.87 (0.33)	<0.001
Men	135 (44.9)	1.00 (0.29)	
Body mass index (kg/m^2^)^a^			
18.5-23.9	130 (43.2)	0.98 (0.30)	0.078
< 18.5	11 (3.7)	0.91 (0.22)	
≥ 24.0	158 (52.5)	0.89 (0.33)	
Education status			
Junior and above	180 (59.8)	0.99 (0.30)	0.001
Primary	121 (40.2)	0.84 (0.33)	
Living status			
With others	280 (93.0)	0.93 (0.32)	0.647
Alone	21 (7.0)	0.90 (0.26)	
Smoking status			
Non-smoker	246 (81.7)	0.92 (0.33)	0.115
Ex- smoker	39 (13.0)	0.94 (0.30)	
Current-smoker	16 (5.3)	1.09 (0.20)	
Drinking status			
Non-drinker	248 (82.4)	0.91 (0.33)	0.06
Ex-drinker	10 (3.3)	0.99 (0.24)	
Current-drinker	43 (14.3)	1.03 (0.25)	
Frequency of exercise^a^			
< 3/ week	54 (17.9)	0.79 (0.32)	<0.001
≥ 3/ week	244 (81.1)	0.96 (0.31)	

^a^Numbers of subjects are not equal to 301 due to missing values in variables

**Table 2:** Six-meter walking speed by physical and mental related comorbid conditions (*n*=301)

	Total	6-meter walking speed (m/sec)	*p-*value for t-test/ ANOVA
	n (%)	Mean (SD)	
**Physical-related comorbid conditions**			
Diabetes mellitus			
Yes	61 (20.3)	0.85 (0.33)	0.029
No	240 (79.7)	0.95 (0.32)	
Hypertension			
Yes	145 (48.2)	0.87 (0.34)	0.004
No	156 (51.8)	0.98 (0.29)	
Heart diseases			
Yes	84 (27.9)	0.84 (0.32)	0.004
No	217 (72.1)	0.96 (0.32)	
Hyperlipidemia			
Yes	88 (29.2)	0.93 (0.37)	0.860
No	213 (70.8)	0.93 (0.30)	
Stroke			
Yes	11 (3.7)	0.68 (0.30)	0.009
No	290 (96.3)	0.94 (0.32)	
Cancer			
Yes	25 (8.3)	0.82 (0.31)	0.073
No	276 (91.7)	0.94 (0.32)	
Gout			
Yes	17 (5.6)	0.93 (0.30)	0.947
No	284 (94.4)	0.93 (0.32)	
Respiration diseases			
Yes	29 (9.6)	0.93 (0.28)	0.988
No	272 (90.4)	0.93 (0.32)	
Lower limb arthritis (hip and knee)^a^			
Yes	58 (19.3)	0.84 (0.23)	0.025
No	240 (79.7)	0.95 (0.34)	
Fall			
Yes	50 (16.6)	0.87 (0.34)	0.151
No	251 (83.4)	0.94 (0.32)	
**Mental -related comorbid conditions**			
Hospital Anxiety and Depression Scale			
Anxiety subscale			
≥ 3	103 (34.2)	0.88 (0.29)	0.061
< 3	198 (65.8)	0.95 (0.33)	
Depression subscale			
≥ 6	30 (10.0)	0.74 (0.29)	<0.001
< 6	271 (90.0)	0.95 (0.32)	

^a^Numbers of subjects are not equal to 301 due to missing values in variables

Table 3 presents the correlations between walking speed, anthropometric measurements, and hand grip strength. Walking speed was associated with age, height, weight, hand grip strength, and muscle mass. Moreover, hand grip strength was significantly associated with total body, trunk, and upper and lower limb muscle mass.

**Table 3:** Correlation matrix between 6-M Walking Speed, anthropometric measurement and hand grip strength

	1	2	3	4	5	6	7	8	9	10
1. 6-M Walking Speed (m/sec)	1									
2. Age (years)	-0.42***	1								
3. Height (cm)	0.32***	-0.23***	1							
4. Weight (kg)	0.12*	-0.21***	0.57***	1						
5. Body mass index (kg/m^2^)	-0.10	-0.09	-0.07	0.78***	1					
6. Hand grip strength (kg)	0.47***	-0.34***	0.65***	0.39***	-0.01	1				
7. Total body muscle mass (kg)	0.26***	-0.22**	0.85***	0.72***	0.23**	0.71***	1			
8. Trunk muscle mass (kg)	0.30***	-0.27***	0.89***	0.65***	0.11	0.71***	0.94***	1		
9. Upper limbs muscle mass (kg)	0.24***	-0.16*	0.77***	0.69***	0.25***	0.64***	0.94***	0.87***	1	
10. Lower limbs muscle mass (kg)	0.20**	-0.16*	0.72***	0.72***	0.31***	0.62***	0.94***	0.78***	0.89***	1

*p < 0.05

**p < 0.01

***p < 0.001

The significant variables shown in Tables 1, 2 and 3 were included in stepwise multiple linear regression. To avoid collinearity, only the muscle mass data of the upper and lower limbs were included in the analysis. Table 4 shows the stepwise multiple linear regression model considering all included participants. Hand grip strength (β = 0.01, standard error [SE] = 0.002, *p* < 0.001) and age (β = −0.15, SE = 0.04, *p* < 0.001) were significantly associated with walking speed, but not with sex. After stratification by sex, hand grip strength was associated with walking speed in both men (β = 0.01, SE = 0.003, *p* < 0.001) and women (β = 0.03, SE = 0.006, *p* < 0.001), while age was associated with walking speed in only women (β = −0.17, SE = 0.06, *p* = 0.003) (Table 5). Accordingly, the association between hand grip strength and walking speed was stable and robust in both sexes. Therefore, hand grip strength can be used as a proxy for the detection of slow walking speed.

**Table 4:** Linear regression analyses for prediction models for the 6-meter walking speed test

	Unadjusted model		Stepwise model			
			Step I		Step II	
	b (se)	*p*	b (se)	*p*	b (se)	*p*
Age (≥ 75 vs < 75)	-0.23 (0.04)	<0.001			-0.15 (0.04)	<0.001
Sex (female vs. male)	-0.13 (0.04)	<0.001				
Education (primary vs junior and above)	-0.15 (0.04)	<0.001				
Exercise (≥ 3/ week vs < 3/ week)	0.17 (0.05)	<0.001				
Height (cm)	0.01 (0.002)	<0.001				
Weight (kg)	0.004 (0.002)	0.047				
Hand grip strength (kg)	0.02 (0.002)	<0.001	0.02 (0.002)	<0.001	0.01 (0.002)	<0.001
Upper limbs muscle mass (kg)	0.07 (0.02)	<0.001				
Lower limbs muscle mass (kg)	0.02 (0.01)	0.002				
Diabetes mellitus (yes vs. no)	-0.10 (0.05)	0.029				
Hypertension (yes vs. no)	-0.11 (0.04)	0.004				
Heart diseases (yes vs. no)	-0.12 (0.04)	0.004				
Stroke (yes vs. no)	-0.26 (0.10)	0.009				
Lower limb arthritis (yes vs. no)	-0.11 (0.05)	0.025				
Depression (yes vs. no)	-0.21 (0.06)	<0.001				
Adjusted R-square			0.20		0.24

**Table 5:** Stepwise multiple linear regression analyses for prediction models for the 6-meter walking speed test stratified by sex

	Women				Men	
	Step I		Step II		Step I	
	b (se)	*p*	b (se)	*p*	b (se)	*p*
Age (≥ 75 vs <75)			-0.17 (0.06)	0.003		
Education						
Exercise						
Height (cm)						
Weight (kg)						
Hand grip strength (kg)	0.04 (0.005)	<0.001	0.03 (0.006)	<0.001	0.01 (0.003)	<0.001
Upper limbs muscle mass (kg)						
Lower limbs muscle mass (kg)						
Diabetes mellitus						
Hypertension						
Heart diseases						
Stroke						
Lower limb arthritis						
Depression						
Adjusted R-square	0.27		0.31		0.12	

**Fig. 1:**
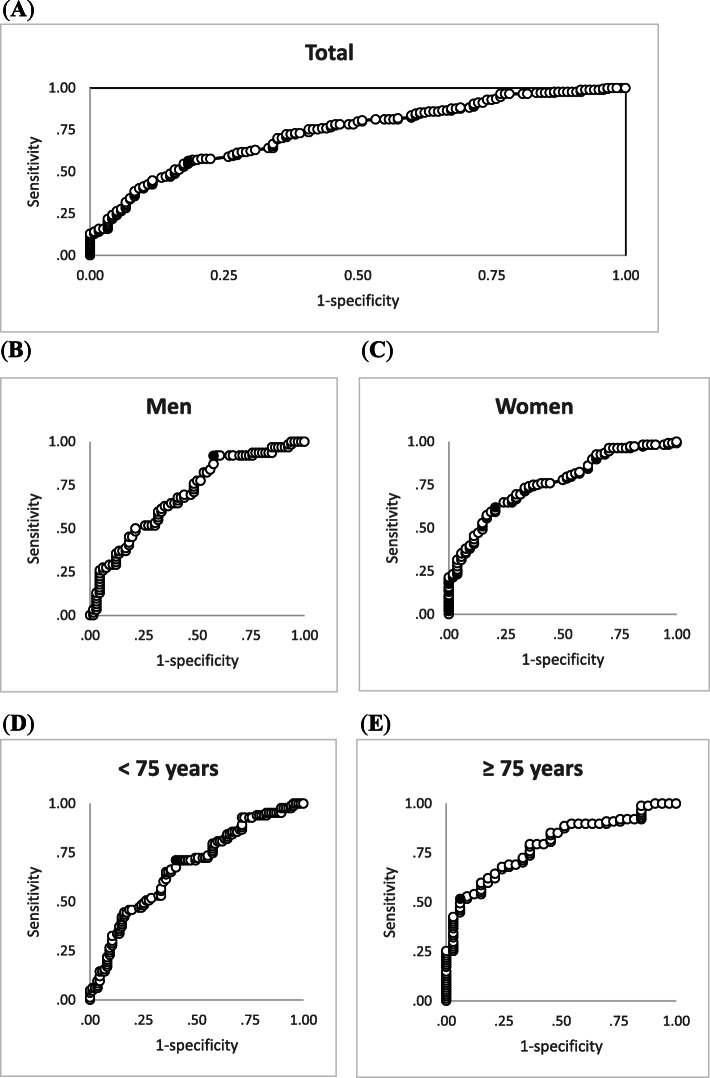
Receiver operating characteristic curve analysis for optimal cutoff values of hand grip strength in detecting slow walking speed. Panel **(A)** total participants; Panel **(B)** men; Panel **(C)** women; Panel **(D)** participants aged < 75 years; Panel **(E)** participants aged ≥ 75 years. Black point indicates the optimal cutoff point”

To develop an instrument for detecting slow walking speed, we adopted two strategies. First, we developed a predicted model according to the final models derived from the previous stepwise multiple linear regression model, in which hand grip strength and age were specified to predict walking speed. Furthermore, slow walking speed was identified according to the latest AWGS criteria. Second, because hand grip strength itself is highly correlated with walking speed, we also used the ROC curve to obtain the optimal cutoff for hand grip strength for detecting slow walking speed. Finally, the performance of these strategies was compared to justify the use of hand grip strength as a single proxy for detecting slow walking speed. The accuracy of the two strategies was comparable (linear prediction model: 68.3 % vs. ROC curve-derived cutoff: 66.9 %). Therefore, we decided to use hand grip strength alone as a proxy for walking speed. To detect slow walking speed, a hand grip strength cutoff value of 19.73 kg for all participants had a sensitivity of 55 %, a specificity of 83 %, an AUC of 0.74, and an accuracy rate of 66.9 %. A hand grip strength cutoff value of 35.10 kg for men had a sensitivity of 92 %, a specificity of 42 %, an AUC of 0.70, and an accuracy rate of 66.4 %. A hand grip strength cutoff value of 17.93 kg in women had a sensitivity of 62 %, a specificity of 80 %, an AUC of 0.76, and an accuracy rate of 67.9 % (Table 6). An area under the ROC curve of 0.7–0.8 had good diagnostic accuracy [28]. Figure 1 showed the ROC curves for optimal cutoff values of hand grip strength in detecting slow walking speed among total participants and subgroups.

**Table 6:** Receiver operating characteristic curve analysis for optimal cutoffs of hand grip strength for detecting slow 6-meter walking speed test

	Hand grip strength (kg)	Sensitivity	Specificity	Area under curve	*p*-value	Accuracy rate (%)
Total	19.73	0.55	0.83	0.74	< 0.001	66.9
Men	35.10	0.92	0.42	0.70	< 0.001	66.4
Women	17.93	0.62	0.80	0.76	< 0.001	67.9
< 75 years	23.65	0.71	0.60	0.68	< 0.001	65.3
≥ 75 years	16.58	0.52	0.94	0.79	< 0.001	63.3

## Discussion

In this study, we found that hand grip strength was significantly associated with walking speed; it was the only variable associated with walking speed in both sexes after considering various sociodemographic, lifestyle, and anthropometric factors in community-dwelling older adults. In addition, we compared the performance of hand grip strength as a single proxy with that of the predicted model in detecting slow walking speed, which showed comparable results. However, a simple cutoff value is more practical in the community. Therefore, we decided to use hand grip strength as a single proxy for detecting slow walking speed.

In this study, the mean participant age was 74.3 years and the prevalence of slow walking speed was 56.5 %. However, in the Aging Study of Pyeongchang Rural Area (ASPRA) in Korea, the mean age was 76 years and the prevalence of slow walking speed was 78.2 % using a cutoff value of < 1.0 m/s [29]. Older mean age of the participants could be an explanation for the higher prevalence reported in the ASPRA. In the Health and Retirement Study conducted in the United States, the mean participant age was 75.4 years and the prevalence of slow walking speed was 53.5 % using a cutoff value of < 0.8 m/s. In our study, if we had used a cutoff value of < 0.8 m/s, fewer participants would have met the criteria for the slow-walking group, which could have led to underestimation of the prevalence of slow walking speed. Our study echoed the 2019 recommendations of the AWGS, which increased the cutoff of slow walking speed to < 1.0 m/s. Therefore, our study indicated that it was necessary to determine the optimal cutoff value for hand grip strength based on the most recent definition of slow walking speed. Furthermore, compared with a similar study in the Turkish population, a hand grip strength cutoff value of 32 kg in men and 22 kg in women was determined among patients visiting an outpatient clinic [22], while a hand grip strength cutoff value of 35.10 kg in men and 17.93 kg in women was implemented among the community-dwelling older adults in our study. Different cutoff values reflect the varying definitions of slow walking speed (< 0.8 vs. < 1.0 m/s) and differing study design.

In the literature, hand grip strength, lower extremity muscle strength, lower extremity muscle power, and calf muscle area have been used to predict slow walking speed [24, 30]. Among these, hand grip strength, lower extremity muscle strength, and upper and lower extremity muscle power have shown comparable predictive ability, but not calf muscle area [24, 30]. Our study revealed that muscle mass was not associated with walking speed, which is consistent with the results of a previous study reporting that muscle strength, but not muscle mass, is independently associated with lower extremity performance [31]. These findings support the use of hand grip strength as a proxy for detecting slow walking speed in community-dwelling older adults owing to its accessibility and availability.

This study also provided age- and sex-specific cutoff values for hand grip strength for the detection of slow walking speed. Men and participants aged < 75 years had higher sensitivity, while women and participants aged ≥ 75 years had higher specificity. Therefore, the performance of hand grip strength in detecting slow walking speed differed among subgroups. Our study also showed that the explanatory power of hand grip strength in predicting walking speed was higher in women than in men (adjusted R^2^: 0.27 vs. 0.12). Therefore, hand grip strength could identify slow walking speed better among men and participants aged < 75 years, while it could identify normal walking speed better among women and participants aged ≥ 75 years. However, sex was not found to be significantly associated with walking speed in our study. In the ROC analysis, after stratification by sex, the optimal cutoff values for detecting slow walking speed did not notably improve the AUC or accuracy rate. However, the sex-specific cutoff values increased the sensitivity. There is also a trade-off between sensitivity and specificity when cutoffs are assigned to a continuous variable. It should be noted that the AWGS does not recommend sex-specific cutoff values for slow walking speed [13]. In a real-world setting, it may be more convenient and practical to adopt uniform, rather than sex-specific, cutoff values; however, from a prevention and public health perspective, sex-specific cutoff values may identify more individuals who are at risk of slow walking speed. Further investigation is needed to determine whether sex-specific cutoff values are essential.

This study had several strengths. First, using face-to-face interviews to obtain information from the participants reduced information bias. Second, various sociodemographic and lifestyle characteristics together with anthropometric measurements were considered in the assessment of the best proxy for the detection of slow walking speed. However, this study also had some limitations. First, participants were limited to community-dwelling older people; therefore, the generalizability of the findings to older people who are institutionalized or who have severe disabilities should be examined further. However, in individuals who are institutionalized or who have severe disabilities, functional decline due to physical conditions or aging tends to be more severe in the lower extremities [32–34]. Muscle strength of the upper extremities, such as hand grip strength, is less affected than that of the lower extremities [32–34]. Second, we used a Camry® dynamometer rather than the standard Jamar® dynamometer (Jamar, Jackson, MI, USA), because it is cheaper, lighter, and easier to use in the community. Additionally, the Camry® dynamometer weighs 1 lbs, while the Jamar® dynamometer weighs 4 lbs. The Camry® dynamometer was validated using the Jamar® dynamometer, and a strong correlation was found [15].

## Conclusions

Our study found hand grip strength to be a clinically useful indicator for the detection of slow walking speed, which is detrimental to the health of older adults. Further investigation is warranted to examine the validity of using hand grip strength as a proxy for walking speed to assess frailty.

## Data Availability

The datasets used and analyzed during the current study are available from the corresponding author on reasonable request.

## Abbreviations

AUC: Area under the curve
AWGS: Asian Working Group for Sarcopenia
ROC: Receiver Operating characteristic
SE: Standard error
